# The *Pontastacus leptodactylus* (Astacidae) Repeatome Provides Insight Into Genome Evolution and Reveals Remarkable Diversity of Satellite DNA

**DOI:** 10.3389/fgene.2020.611745

**Published:** 2021-01-21

**Authors:** Ljudevit Luka Boštjančić, Lena Bonassin, Lucija Anušić, Leona Lovrenčić, Višnja Besendorfer, Ivana Maguire, Frederic Grandjean, Christopher M. Austin, Carola Greve, Alexander Ben Hamadou, Jelena Mlinarec

**Affiliations:** ^1^Division of Molecular Biology, Department of Biology, University of Zagreb, Zagreb, Croatia; ^2^Division of Zoology, Department of Biology, University of Zagreb, Zagreb, Croatia; ^3^Laboratoire Ecologie Biologie des Interactions-UMR CNRS 7267, University of Poitiers, Poitiers, France; ^4^Centre of Integrative Ecology, School of Life and Environmental Sciences Deakin University, Geelong, VIC, Australia; ^5^LOEWE Centre for Translational Biodiversity Genomics (LOEWE-TBG), Frankfurt, Germany

**Keywords:** FISH, genome size, interstitial telomeric repeats, karyotype, narrow-clawed crayfish, (peri)centromeric heterochromatin

## Abstract

*Pontastacus leptodactylus* is a native European crayfish species found in both freshwater and brackish environments. It has commercial importance for fisheries and aquaculture industries. Up till now, most studies concerning *P. leptodactylus* have focused onto gaining knowledge about its phylogeny and population genetics. However, little is known about the chromosomal evolution and genome organization of this species. Therefore, we performed clustering analysis of a low coverage genomic dataset to identify and characterize repetitive DNA in the *P. leptodactylus* genome. In addition, the karyogram of *P. leptodactylus* (2*n* = 180) is presented here for the first time consisting of 75 metacentric, 14 submetacentric, and a submetacentric/metacentric heteromorphic chromosome pair. We determined the genome size to be at ~18.7 gigabase pairs. Repetitive DNA represents about 54.85% of the genome. Satellite DNA repeats are the most abundant type of repetitive DNA, making up to ~28% of the total amount of repetitive elements, followed by the Ty3/*Gypsy* retroelements (~15%). Our study established a surprisingly high diversity of satellite repeats in *P. leptodactylus*. The genome of *P. leptodactylus* is by far the most satellite-rich genome discovered to date with 258 satellite families described. Of the five mapped satellite DNA families on chromosomes, PlSAT3-411 co-localizes with the AT-rich DAPI positive probable (peri)centromeric heterochromatin on all chromosomes, while PlSAT14-79 co-localizes with the AT-rich DAPI positive (peri)centromeric heterochromatin on one chromosome and is also located subterminally and intercalary on some chromosomes. PlSAT1-21 is located intercalary in the vicinity of the (peri)centromeric heterochromatin on some chromosomes, while PlSAT6-70 and PlSAT7-134 are located intercalary on some *P. leptodactylus* chromosomes. The FISH results reveal amplification of interstitial telomeric repeats (ITRs) in *P. leptodactylus*. The prevalence of repetitive elements, especially the satellite DNA repeats, may have provided a driving force for the evolution of the *P. leptodactylus* genome.

## Introduction

Freshwater crayfish constitute a monophyletic group of over 640 described species, arranged into four families: Astacidae, Cambaridae, Cambaroididae, and Parastacidae (Crandall and De Grave, 2017). These species are distributed across all but the Antarctic continent, the Indian subcontinent, and African mainland, with centers of diversity in the southeastern Appalachian Mountains in the North America and southeastern Australia (Crandall and Buhay, 2008). The Northern (Astacidae, Cambaroididae, and Cambaridae) and Southern (Parastacidae) hemisphere families form deeply divergent reciprocally monophyletic clades (Bracken-Grissom et al., 2014). The crayfish species of the family Astacidae belong to four genera of which *Pacifastacus* is native to North America, while *Astacus, Pontastacus*, and *Austropotamobius* are native to the European continent (Crandall and De Grave, 2017). In the last decades numbers and sizes of native European crayfish populations have been in decline due to climate change, degraded water quality, negative anthropogenic pressure on freshwater habitats, and the introduction of alien invasive crayfish species and their pathogens (e.g., *Aphanomyces astaci*) (Holdich et al., 2009; Kouba et al., 2014). One of the native European crayfish species is *Pontastacus leptodactylus* (Eschscholtz, 1823), found both in freshwater and brackish environments with a nowadays distribution encompassing Europe, eastern Russia, and the Middle East (Kouba et al., 2014). Up till now, the majority of studies on this species have focused on morphology, phylogeny and population genetics (Maguire and Dakić, 2011; Akhan et al., 2014; Maguire et al., 2014; Gross et al., 2017; Khoshkholgh and Nazari, 2019). Analyses of phylogenetic relationships among *P. leptodactylus* populations, using mtDNA, revealed three well-supported divergent lineages; one distributed in Europe (Croatia, Bulgaria, Poland, and Turkey) (European lineage *sensu* Maguire et al., 2014), another in Asia (Armenia, Russia) (Asian lineage *sensu* Maguire et al., 2014), and the third endemic to Turkey (Clade III *sensu* Akhan et al., 2014). While genomic information has started to accumulate for North American and Australian species (Gutekunst et al., 2018; Tan et al., 2020; Van Quyen et al., 2020), so far few studies have focused on cytogenetic and genome organization of European freshwater crayfish species (Mlinarec et al., 2011, 2016), and therefore the general aim of this study was to increase knowledge on genome evolution and diversity focusing on repetitive DNAs in *P. leptodactylus*.

The majority of animal and plant genomes contain a substantial portion of repetitive DNA, collectively referred to as the repeatome of a species, which is considered largely responsible for genome size variation. The repeatome is comprised of dispersed (DNA transposons and retrotransposons) and tandemly arranged sequences (such as nuclear ribosomal RNA genes and satellite DNAs) (Garrido-Ramos, 2017). Satellite DNAs (satDNAs) are organized in large tandem arrays of highly repetitive non-coding short sequences. SatDNAs are one of the most rapidly evolving DNAs in the genome (Garrido-Ramos, 2017). Their evolution is mainly marked by amplification and homogenization processes (both decreasing divergence) and point mutations (increasing divergence) (Ruiz-Ruano et al., 2019). Considering the differences in the size of the repeating units, satDNAs are classified into microsatellites (repeat units <10 bp), minisatellites (repeat units in the range 10–100 bp), and conventional satellites (repeat units larger than 100 bp) (Garrido-Ramos, 2017). Conventional satellites are found specifically at pericentromeric and subtelomeric locations of the chromosomes, but might be found occupying interstitial positions of the chromosomes constituting heterochromatin segments (HSs) (Garrido-Ramos, 2017). The satDNAs perform functions in the regulation of gene expression and play an important structural role in the vital functions including among others, chromosome segregation and the preservation of genetic material (Blackburn, 2005; Louis and Vershinin, 2005; Riethman et al., 2005; Kuo et al., 2006).

The characterization of repetitive DNAs from poorly characterized genomes or species lacking a reference genome can be a challenging task (Ávila Robledillo et al., 2018). Up to now, only a few satDNAs have been reported in crustaceans, manly using traditional methods such as centrifugation through sequential CsCl gradients (Chambers et al., 1978; Wang et al., 1999). Today, repetitive DNAs can now be analyzed more easily owing to the recent advances in next generation sequencing (NGS) and high-throughput *in silico* analysis of the information contained in the NGS reads (Weiss-Schneeweiss et al., 2015; Ruiz-Ruano et al., 2019). Development of the RepeatExplorer software tool allows for *de novo* repeat identification using analyses of short sequences, randomly sampled from the genome (Novák et al., 2010, 2013). The Tandem Repeat Analyzer (TAREAN) further improved the RepeatExplorer pipeline allowing for the automatic identification and reconstruction of monomer sequences for each satDNA family in the species, collectively referred to as satellitome (Novák et al., 2017).

Decapod crustaceans present an attractive study model due to the existence of polyploidy, a large quantity of AT-rich HSs as well as the adaptation to a broad range of environments (Mlinarec et al., 2011; Martin et al., 2015; Tan et al., 2019). However, the majority of crustaceans have been poorly investigated at the genomic and cytogenomic level (Tan et al., 2020; Van Quyen et al., 2020). To a large extent, this is reflective of the fact that decapod crustaceans, and freshwater crayfish in particular, have a low mitotic index, a high diploid chromosome number, small chromosomes, and highly repetitive genomic elements (Tan et al., 2004, 2019, 2020; Mlinarec et al., 2011; Gutekunst et al., 2018; Van Quyen et al., 2020). Therefore, cytogenetic studies on freshwater crayfish species are rare, often limited to the report of chromosome number and structure, with very few reports on molecular cytogenetics (Tan et al., 2004; Indy et al., 2010; Scalici et al., 2010; Mlinarec et al., 2011, 2016; Kostyuk et al., 2013; Salvadori et al., 2014) (Table 1).

**Table 1:** Chromosomal and cytogenetic characteristics of freshwater crayfish species of families Astacidae, Cambaridae, and Parastacidae.

**Family/species**	**2*n***	**45S rDNA**	**Karyotype formula**	**IHSs**	**Reference**
**Family Astacidae**					
*Astacus astacus*	176	2	52m+35sm+1a	22 pairs of chromosomes	Mlinarec et al., 2011
Pontastacus leptodactylus*^(1)^	180	2		6 pairs of chromosomes	Mlinarec et al., 2011
Pontastacus leptodactylus*^(2)^	180	1	75m+14sm+1sm/m	10 pairs of chromosomes	This study
*Austropotamobius torrentium*	176	2	76m+11sm+1a		Mlinarec et al., 2016
*Austropotamobius pallipes*	176	2	76m+11sm+1a		Mlinarec et al., 2016
*Pacifastacus leniusculus*	376				Niiyama et al., 1962
**Family Cambaroididae**					
Cambaroides japonicus	194				Komagata and Komagata, 1992
**Family Cambaridae**					
*Procambarus clarkii*	188	2			Salvadori et al., 2014
*Procambarus llamasi*	120		120t		Indy et al., 2010
*Procambarus digueti*	102		35M+15m+1st		Diupotex Chong et al., 1997
*Procambarus alleni*	188				Martin et al., 2015
*Procambarus fallax*	184				Martin et al., 2015
Procambarus virginalis*^(3)^	276		171m+39sm+3st+63t		Martin et al., 2015
*Faxonius virilis*	200				Fasten, 1914
*Faxonius immunis*	208				Fasten, 1914
**Family Parastacidae**					
*Cherax destructor*	188		70m+42sm+48st+28t		Scalici et al., 2010
*Cherax quadricarinatus*	200		33m+25sm+14st+28t		Tan et al., 2004

*2n, diploid chromosome number; 45SrDNA, number of 45S rDNA loci; IHSs, number of homologous chromosome pairs possessing probable interstitial heterochromatic segments. m, metacentric; ms, metacentric-submetacentric; sm, submetacentric; a, acrocentric; t, telocentric; st, subtelocentric chromosome. Species classification according to Crandall and De Grave (2017)*.

*(1) *Euroasian lineage*.

*(2) *Asian lineage I*.

*(3) *autotriploid species*.

Keeping in mind the lack of research in the field for European freshwater crayfish, this study aims to: (i) identify and characterize repetitive sequences in the *P. leptodactylus* genome in order to get better insight into genome organization and evolution of this species, and (ii) analyze the chromosomal distribution patterns of major tandem repetitive DNA families to contribute with the chromosome organization and evolution. In addition, *COI* barcoding was used to place the samples used in this study within the context of patterns of diversity to determine the phylogenetic placement of *P. leptodactylus* individuals from Lake Maksimir.

## Materials and Methods

### Samples and DNA Extraction

Seven individuals (four males and three females) of narrow-clawed crayfish *Pontastacus leptodactylus* (Eschscholtz, 1823) were collected from the Third Maksimir Lake (Zagreb, Croatia); 45.82972°N 16.02056°E.

One pereiopod from each individual was removed and stored in 96% ethanol at 4°C until DNA extraction. Genomic DNA was isolated from muscle tissue using the GenElute Mammalian Genomic DNA Miniprep kit (Sigma-Aldrich, St. Louis, MO) following the manufacturer's protocol and stored at −20°C.

### DNA Barcoding and Phylogenetic Network Reconstruction

Mitochondrial cytochrome oxidase subunit I (*COI*) barcode region was amplified and sequenced from genomic DNA of two individuals taken from Lake Maksimir using primer pairs LCO-1490 (5′-GGTCAACAAATCATAAAGATATTGG-3′) and HCO-2198 (5′-TAAACTTCAGGGTGACCAAAAAATCA-3′) described in Folmer et al. (1994). PCR reaction conditions and purification of PCR product followed the protocols described in Maguire et al. (2014). Sequencing of purified PCR products was performed by Macrogen Inc. (Amsterdam, Netherlands). Phylogenetic analysis included a total of 129 *COI* gene sequences of which 127 were downloaded from GenBank (accession KX279350), while the other two were obtained from Lake Maksimir individuals obtained in this study (Supplementary Table 1). Sequences were edited using SEQUENCHER 5.4.6 (Gene Codes Corporation, Ann Arbor, MI, USA) and aligned using MAFFT (Katoh and Standley, 2013). Sequences were collapsed to unique *COI* haplotypes using the software DnaSP 6.12.03 (Rozas et al., 2017). A median joining network was constructed on *COI* haplotype dataset using PopArt (Bandelt et al., 1999) to visualize non-hierarchical haplotype relationships and their geographical distribution. Sites containing ambiguities were excluded from network reconstruction. This approach is recommended as a standard for cytogenetic studies as it links karyotypes with DNA barcodes (Lukhtanov and Iashenkova, 2019).

### Flow Cytometry Analysis

The genome size was estimated following a flow cytometry protocol with propidium iodide-stained nuclei described in Hare and Johnston (2011). Different tissue (tail muscle, vascular tissue, and gills) of −80°C frozen adult samples of *P. leptodactylus* and neural tissue of the internal reference standard *Acheta domesticus* (female, 1C = 2Gb) was each mixed and chopped with a razor blade in a petri dish containing 2 ml of ice-cold Galbraith buffer. The suspension was filtered through a 42-μm nylon mesh and stained with the intercalating fluorochrome propidium iodide (PI, Thermo Fisher Scientific) and treated with RNase II A (Sigma-Aldrich), each with a final concentration of 25 μg/ml. The mean red PI fluorescence of stained nuclei was quantified using a Beckman-Coulter CytoFLEX flow cytometer with a solid-state laser emitting at 488 nm. Fluorescence intensities of 5000 nuclei per sample were recorded. We used the CytExpert 2.3 software for histogram analyses. The total quantity of DNA in the sample was calculated as the ratio of the mean fluorescence signal of the 2C peak of the stained nuclei of the crayfish sample divided by the mean fluorescence signal of the 2C peak of the stained nuclei of the reference standard times the 1C amount of DNA in the reference standard. Three individuals were scored to produce biological replicates. For one individual we prepared different tissues to make sure that we have not used polyploid tissue. The genome size is reported as 1C, the mean amount of DNA in Mb in a haploid nucleus.

### Next Generation Sequencing, Data Pre-processing, and Clustering Analysis

Raw Illumina pair-end reads 150 bp long obtained from low coverage DNA-seq experiments on *Pontastacus leptodactylus* are available from the European Nucleotide Archive (NGS run accession: SRR7698976). After the quality filtering (quality cut-off value: 10 according to Novák et al., 2020b; percent of bases in sequence that must have quality equal to/higher than the cut-off value: 95 and filtered against a costomized database containing *P. leptodactylus* mitochondrial sequences), the reads were subjected to similarity-based clustering analysis using RepeatExplorer2 (Novák et al., 2010, 2013). We used a subset of reads (2 × 125,000) representing coverage of 0.002×. Genome coverage was calculated as follows: coverage = (*r* × l)/g, where r corresponds to number of reads used in our analysis, l to read length and g to haploid genome size of *P. leptodactylus*. The clustering was performed using the default settings of 90% similarity over 55% of the read length. To confirm the results obtained through the RepeatExplorer pipeline, reconstruction of monomer sequences of individual satellite DNA families was performed using TAREAN analysis, specific for identification of satellite DNA repeats (Novák et al., 2017).

### Repeat Classification

Repeat cluster classification of the top 0.01% clusters identified in comparative analysis was implemented in RepeatExplorer through which similarity searches with DNA and protein databases. After *de novo* identification of contigs that make up repetitive elements in RepeatExplorer, contigs were further classified using two homology-based approaches applied in LTRClassifier (Monat et al., 2016), specific for LTR retrotransposons, and Censor (Jurka et al., 1996) for all repetitive elements. This was followed by manual examination of individual clusters graph shapes, similarity searches using BLASTN and BLASTX against public databases (https://blast.ncbi.nlm.nih.gov/Blast.cgi), inspection for the presence of sub-repeats using program dotmatcher (https://www.bioinformatics.nl/cgi-bin/emboss/dotmatcher) with parameters specific to individual monomer length (10% of length as window size and sequence specific similarity cut off), for the final manual annotation and quantification of repeats.

Putative satellite repeats were identified based on the properties of cluster graphs obtained by similarity-based clustering of low coverage genome sequencing Illumina reads, as implemented in the TAREAN pipeline (Novák et al., 2017). All satellite repeats with an abundance exceeding 0.1% of the *P. leptodactylus* genome were subjected to detailed sequence analysis (Supplementary Table 2). This analysis focused on AT content, genomic abundance, and presence of telomeric (TTAGG)_*n*_ repeats and detection of sequence similarities (Supplementary Table 2). Individual satellite DNA clusters were further classified into the satellite groups via h-CD-HIT-EST (Fu et al., 2012) in two consecutive runs, with sequence identity cut-off set at 90% followed by 80% cut-off. Algorithm parameters were kept at default value. Furthermore, we classified tandem repeats as minisatellites (10–100 bp) and conventional satellites (>100 bp) depending on the monomer size (Garrido-Ramos, 2017).

To explore the relation between the repeat length and the %GC of satellite DNA we first performed Shapiro–Wilk's test to access the normality of both length and the %GC variable. Because the length variable did not follow normal distribution, we used non-parametric Spearman's rank correlation test to access correlation between two variables. Bioinformatic and statistical analysis were conducted in the R software environment (R Core Team, 2016).

### Primer Design, PCR Amplification, and Cloning of Satellite DNA Families

From the *P. leptodactylus* reference monomers, outward facing primers were designed (Table 2, Supplementary Figure 1). Specific primer pairs have been used for amplification of satellite DNA probes for FISH. All PCRs were performed using GoTaq® Green Master Mix (Promega, Madison, WI, USA): 1X GoTaq® Green Master Mix, 10 pmol of each primer (Macrogen, Amsterdam, The Netherlands) and 1 μl of template DNA (16 ng), in a 50 μl final reaction volume. PCR program consisted of 35 cycles, each with 1 min denaturation at 95°C, 10 s annealing at 56°C, 1 min extension at 72°C, and a final extension of 20 min.

**Table 2:** Characterization of selected satellite DNA families in *P. leptodactylus*.

**Satellite family**	**Genome abundance (%)**	**Monomer (bp)**	**GC (%)**	**Localization**	**Primer Sequence (5^**′**^-3^**′**^)**
PlSAT1–21	10.91	21	47.62	Interstitial	F-AGTTTCAATCGTCCCTGCTG
					R-TCAGCAGGGACGATTGAAAC
PlSAT3–411	1.29	411	26.76	(Peri)centromeric	F-TGTCTATTTTCCGTATATTGTAATGA
					R-ATCAACCATTTGCATTTCGTTC
PlSAT6–70	0.40	70	35.71	Interstitial	F-GACATGTTTTACATTAGACTTGTGA
					R-TATATGTGCCTGCAAGGTAAGT
PLSAT7–134	0.35	134	29.10	Interstitial	F-GGCAAGCCCAATTGGGTCTGA
					R-TCCGTAACGAAAGTAGAC
PLSAT14–79	0.17	79	44.30	Subtelomeric, interstitial, (peri)centromeric, the whole arm	F-GGTCAGTAAGCTATTGTGTGT
					R-CAACCTATGGAAGGTTATTAAGG

*Repeat unit lengths, G+C content (%), abundances (%), chromosomal localization, and primer pairs used for satellite repeat amplification*.

The sequences of the amplified monomers were verified by cloning of the PCR product into pGEM-T Easy vector according to the manufacturer's instruction (Promega, Madison, WI, USA). Amplicons were extracted and purified using ReliaPrep™ DNA Clean-Up and Concentration System and cloned into pGEM-T Easy vector according to the manufacturer's instruction (Promega, Madison, WI, USA). The individual clones (from one to four per sample) were sequenced by Macrogen (Amsterdam, The Netherlands).

### Preparation of Chromosome Spreads, Chromosome Measurements, and Idiogram Reconstruction

Four adult males (*m* = 17.01, 16.10, 32.21, and 16.27 g) were used for the cytogenetic study. Chromosome spreads were prepared according to the method described in Mlinarec et al. (2011). Individual chromosomes in karyotype were measured using LEVAN plug-in (Sakamoto and Zacaro, 2009) for the program ImageJ (Schneider et al., 2012) to obtain the relative chromosomal length (RCL) data. RCL were then imported into the RIdiogram package (Hao et al., 2020) of R programing environment for the ideogram reconstruction. Idiogram was further modified in the Inkscape vector graphics software (Inkscape Project, 2020) to include the 45S rDNA and DAPI-positive bands.

### Fluorescence *in situ* Hybridization (FISH)

The 2.4 kb *Hind*III fragment of the partial 18S rDNA and ITS1 from *Cucurbita pepo*, cloned into the pUC19 vector, was used as the 45S rDNA probe (Torres-Ruiz and Hemleben, 1994). Telomeric DNA was generated by PCR amplification in the absence of template using primers (TTAGG)_4_ and (CCTAA)_4_ according to Ijdo et al. (1991). Probes used to map satDNAs in the chromosomes were DNA fragments cloned into the plasmid vector. Plasmids containing the monomer sequence were directly labeled with either Aminoallyl-dUTP-Cy3 (Jena Bioscience GmbH, Jena, Germany) or Green-dUTP (Abbott Molecular Inc., USA) using Nick Translation Reagent Kit according to the manufacturer's instructions (Abbott Molecular Inc., USA) with some modifications: Plasmid DNA (700 ng) was labeled in a total volume of reaction of 25 μl using 2.5 μl of enzyme mixture for 6 h at 15°C. FISH was performed according to Mlinarec et al. (2019) with slight modification: chromosome preparations were denatured at 72°C for 5 min after applying the hybridization mix. The preparations were mounted in Dako Fluorescence Mounting Medium (Dako North America Inc., USA) and stored at 4°C overnight. Signals were visualized and photographs captured using an Olympus BX51 microscope, equipped with a cooled CCD camera (Olympus DP70). Single channel images were overlaid and contrasted using Adobe Photoshop 6.0 with only those functions that apply to the whole image. An average of 10 well-spread metaphases was analyzed per each individual.

### Accession Codes

Cloned sequences of satellite repeats were deposited in genBank under accession numbers MW044674 for PlSAT1-21, MW044678 for PlSAT3-411, MW044675 for PlSAT6-70, MW044677 for PlSAT7-134, and MW044676 for PlSAT14-79. *COI* gene sequences were deposited in GenBank under accession numbers MW045515 for Hap1 and MW045516 for Hap2.

## Results

### DNA Barcoding and Phylogenetic Network Reconstruction

Phylogenetic tree was constructed to place samples used in this study within the context of patterns of diversity across the range of *P. leptodactylus*. Final alignment consisted of *COI* barcode sequences 487 bp long and included 91 unique haplotypes from across 10 countries. Haplotype relatedness and geographical haplotype distribution is presented in the Supplementary Figure 2. Three distinct lineages were observed in the median joining network, separated by 8–24 mutational steps. DNA barcoding showed that the samples from the lake Maksimir (Zagreb, Croatia) belong to the Asian lineage *sensu* Maguire et al. (2014) and formed two haplotypes (Hap 1 and Hap 2) closely related to haplotypes from Armenia.

### *Pontastacus leptodactylus* Karyotype and Genomic Organization of 45S rDNA and Telomeric (TTAGG)*_*n*_* Repeats

**Figure 1:**
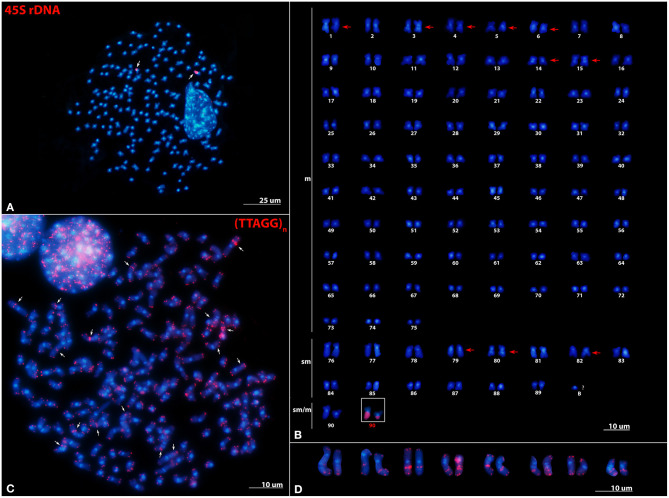
Distribution of 45S rDNA and telomeric repeats on metaphase chromosomes of *Pontastacus leptodactylus*. **(A)** Mitotic metaphase and **(B)** karyogram of *P. leptodactylus* after FISH with 45S rDNA probe (red signals). m, metacentric chromosomes; sm, submetacentric chromosomes; sm/m, submetacentric/metacentric heteromorphic chromosome pair. Arrows point to interstitial HSs. 45S rDNA bearing heteromorphic chromosome pair is framed. **(C)** Mitotic metaphase of *P. leptodactylus* after FISH with telomeric repeats (TTAGG)_n_ (red signals). ITRs are marked with arrows. **(D)** Eight chromosome pairs possessing ITRs. Chromosomes are counterstained with DAPI. Scale bar = 10 μm.

The karyogram of *P. leptodactylus* (2*n* = 180) is presented here for the first time (Figures 1A,B). The karyotype consists of 75 metacentric, 14 submetacentric, and 1 submetacentric/metacentric heteromorphic chromosome pair. Thus, the proposed diploid formula is 2*n* = 75m+14sm+1sm/m. The probable HSs revealed after DAPI staining were found in the (peri)centromeric region of all chromosome pairs as well as in the intercalary regions of 10 chromosome pairs. FISH performed with the 45S rDNA probe revealed two signals positioned on the entire longer arm of the submetacentric/metacentric heteromorphic chromosome pair (Figures 1A,B). Chromosome size and morphology of each chromosome pair within the complement is presented in Supplementary Table 3, while idiogram with position DAPI-positive bands and 45S rDNA loci is presented in Supplementary Figure 3.

FISH experiments using the probe (TTAGG)_*n*_ revealed strong and consistent signals in the terminal ends of both chromosomal arms of all *P. leptodactylus* chromosomes. The telomeric probe also hybridized to interstitial regions (ITRs) of eight chromosome pairs (Figures 1C,D). The ITR signals were of different sizes and intensity and the majority of ITR signals were more intense than the signals in the terminal chromosome ends. All ITRs were devoid of microscopically recognizable heterochromatic regions and did not co-localize with 45S rDNA loci.

### *Pontastacus leptodactylus* Repeatome Characterization and Identification of Tandem Repeats

**Figure 2:**
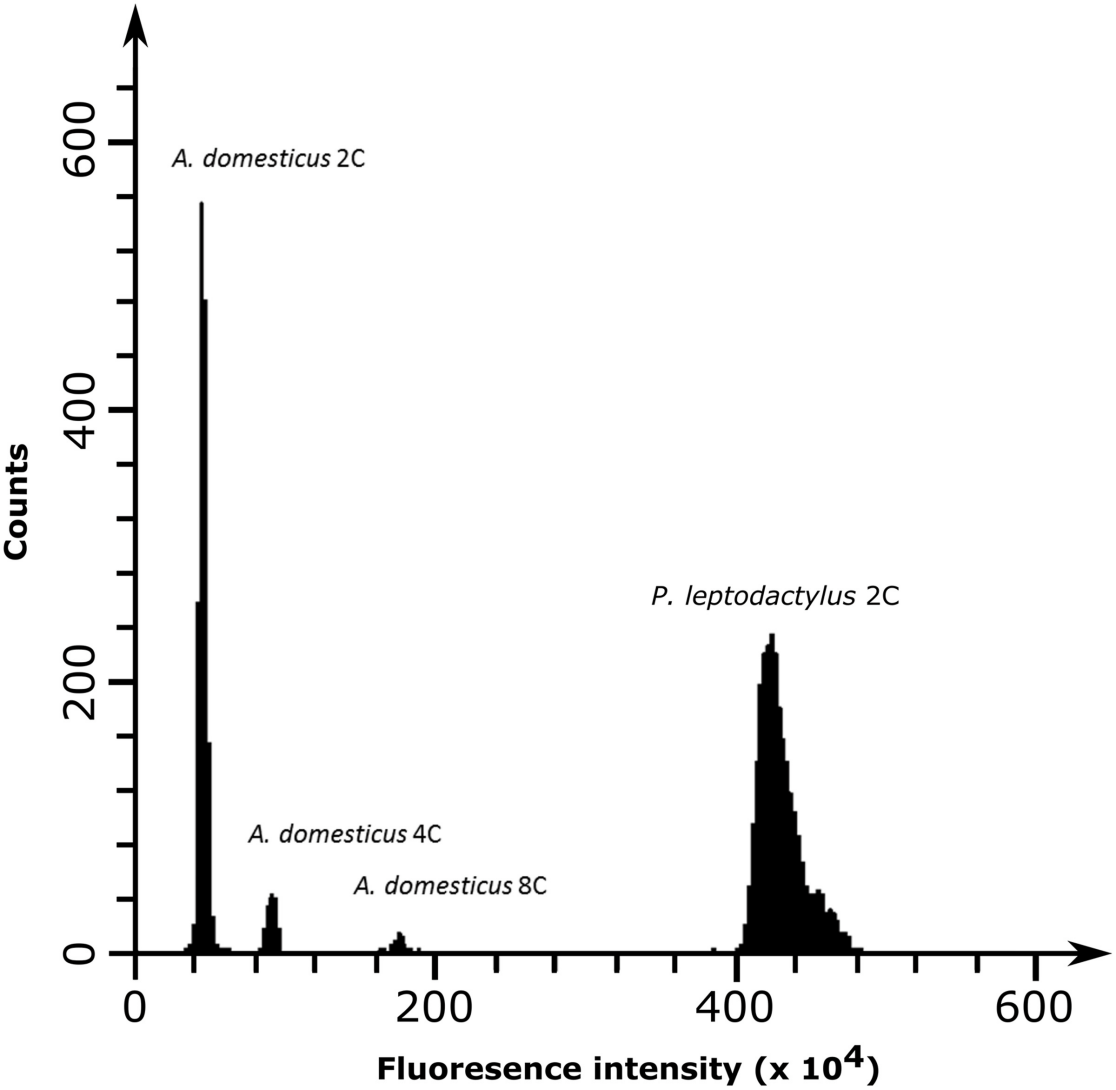
Flow cytometry histograms of neural tissue from house cricket *Acheta domesticus* 2C (first peak), *A. domesticus* 4C (second peak), *A. domesticus* 8C (third peak), and vascular tissue from *P. leptodactylus* (fourth peak) obtained by PI fluorescence dye excitation and counts representing the cell population.

The genome size of *P. leptodactylus* was measured in three individuals from a single population. Results showed that the average 1C DNA value was 18.7 Gbp (Figure 2). Clustering of 2× 125,000 paired-end reads resulted in 19,092 clusters. The nuclear repetitive DNA constituted 54.85% of the genome (Table 3). Of all the repetitive elements, 84.1% were classified to the known repetitive element groups (belonging to 37 major categories), while 4.48% remained unclassified as “other.” Satellite repeats were the most abundant elements, representing 27.52% of the genome, of which minisatellites (10–100 bp) comprised 24.7%, while conventional satellites (>100 bp) comprised 2.87% of the genome. Transposable elements (TEs) contributed 22.67% to the *P. leptodactylus* nuclear genome. Repeats classified as LTR retrotransposons represented the major fraction of the TEs of *P. leptodactylus*, comprising 15.32% (71 clusters) of nuclear DNA, followed by DIRS, LINE, and Penelope elements that comprised 3.57% (4 clusters), 2.23% (33 clusters), 1.00% (2 clusters) of nuclear DNA, respectively. LTR retrotransposons were mostly represented by Ty3/*gypsy* elements (14.95%, 55 clusters), followed by Ty1/*copia* (0.1%, 4 clusters), BEL (0.05%, 2 clusters), and ERV (0.03%, 1 cluster). DNA transposons constituted 0.51% (23 clusters) of the nuclear genome, with Helitrons as the most abundant (0.15%, 6 clusters). Ribosomal RNA genes (45S rDNA) represented 0.01% (1 cluster) of the genome (Table 3).

**Table 3:** Major types of repetitive DNA in *P. leptodactylus* (classification according to Wicker et al., 2007).

**Categories/Superfamilies**	**Abundance (%)**	**Clusters (n)**
Satellites	27.57	258
*Minisatellites*	24.70	240
*Satellites*	2.87	18
**Class I (retrotransposons)**		
LTR elements	15.32	71
*Ty3/gypsy*	14.95	55
*Ty1/copia*	0.10	4
*BEL*	0.05	2
*ERV*	0.03	1
*Integrated virus DNA*	0.19	9
DIRS	3.57	4
Penelope	1.00	2
LINE	2.23	33
*R1*	0.04	2
*R2*	0.03	2
*RTE*	0.01	1
*RTEX*	0.03	1
*Jockey*	0.10	7
*I*	0.21	1
*L1*	0.02	1
*Ingi*	0.05	1
*CRE*	0.01	1
CR1	1.32	6
CR2	0.03	2
*Nimb*	0.03	2
*Kiri*	0.02	1
*Daphne*	0.01	1
*Unclassified LINE*	0.32	4
SINE	0.07	1
**Class II (DNA transposons)—Subclass 1**		
TIR	0.27	14
*Mariner*	0.05	4
*hAT*	0.05	2
*CACTA*	0.03	1
*Harbinger*	0.03	1
*piggyBAC*	0.03	1
*Dada*	0.02	1
*Ginger2*	0.02	1
*Sola*	0.02	1
*Ginger3*	0.01	1
*Transisb*	0.01	1
**Class II (DNA transposons)-Subclass 2**		
Helitron	0.15	6
Polinton	0.09	3
rDNA	0.01	1
Unclassified repetitive	4.48	97
Total repetitive DNA	54.85	490

**Figure 3:**
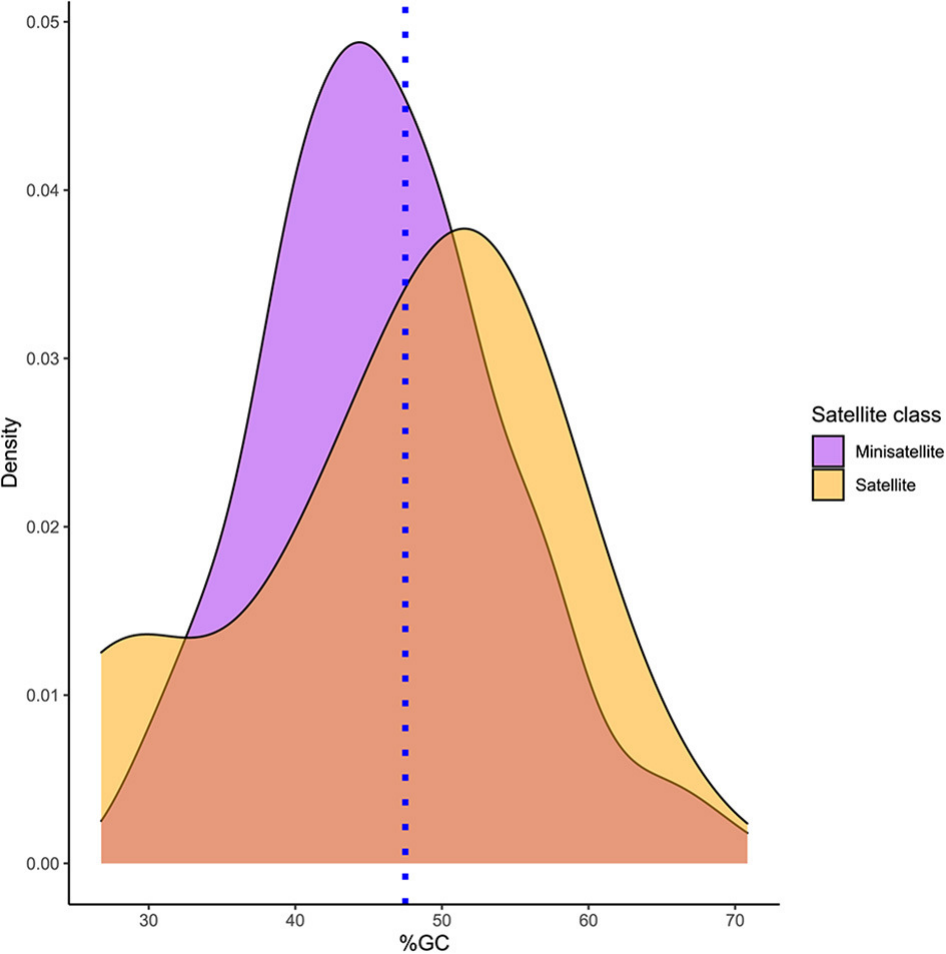
Density plot representing the %GC distribution of *P. leptodactylus* minisatellite and satellite repeats. Blue line represents average %GC percentage of *P. leptodactylus* reported from the NGS reads.

Based on the RepeatExplorer pipeline, 258 satellite DNA families have been identified. Satellite DNA families have been designated as PlSAT1-21, through PlSAT258-57 (stands for *Pontastacus leptodactylus* satellite 1 through to 258 in decreasing genomic abundance, with the respective monomer length separated by a dash; Supplementary Table 2). Their unit lengths ranged from 14 to 664 bp (median value 59 bp; Supplementary Table 2). The distribution of the lengths was biased due to the predominance of short satellite repeats, with more than half (240) being classified as minisatellites. The A+T content of the consensus satDNA sequences varied between 29.17 and 73.14% among the families, with a median value of 54.34%, which indicated a slight bias toward A+T rich satellites. Spearman's rank correlation test showed no significant correlation between satellite length and A+T content (*p*-value: 0.368, correlation coefficient: 0.056) (Figure 3). Only one monomer of the perfect telomeric sequence motif (TTAGG/CCTAA) was present within the consensus sequence of 13 satellite elements, while monomers of other satDNAs contained no telomeric sequence motifs. Based on BLAST searches the satDNA sequences showed no similarity with any other DNA sequence deposited in non-redundant databases. Supplementary Table 2 shows the reconstruction of representative monomer sequences for each satDNA family. Genomic abundance of satellite DNAs ranged from 0.01% up to 10.91% of the genome (Supplementary Table 2). SatDNA family PlSAT1-21 showed the highest abundance (10.91%), followed by PlSAT2-21 (3.79%) and PlSAT3-411 (1.29%).

### Detailed Characterization and Chromosomal Localization of PlSAT3-411, PlSAT6-70, PlSAT7-134, and PlSAT14-79 Satellite DNA Families

Five satellite DNA families, PlSAT1-21, PlSAT3-411, PlSAT6-70, PlSAT7-134, and PlSAT14-79 were selected for further analysis (Table 2, Supplementary Figures 1, 4–6). Firstly, to confirm their tandem arrangement the predicted monomer sequences of selected satellite DNA families have been validated by performing PCR with *P. leptodactylus* genomic DNA as a template using primers designed to face outwards from the reconstructed monomer consensus (Table 2, Supplementary Figure 1). In this arrangement, the amplification can occur only between the primer pairs located in adjacent tandemly repeated arrays. All five putative repeats tested using this assay produced the expected amplification products, and their cloned sequences (from one to four per satellite) matched the predicted consensus with 82–100% similarity. The lowest similarity (82%) was observed between cloned and predicted consensus PlSAT7-134 repeat, while the other four satellite families exhibited 95–100% similarity between cloned and predicted consensus sequence. We selected the one with the highest identity to the reference monomer as the probe for subsequent hybridizations.

Dot plot analysis of PlSAT1-21, PlSAT3-411, PlSAT6-70, PlSAT7-134, and PlSAT14-79 did not reveal any consecutive tandem sub-repeats, although multiple poly-A and poly-T repetitions were observed in GC poor satellite repeat families PLSAT3-411 and PLSAT7-134 (Supplementary Figure 6).

**Figure 4:**
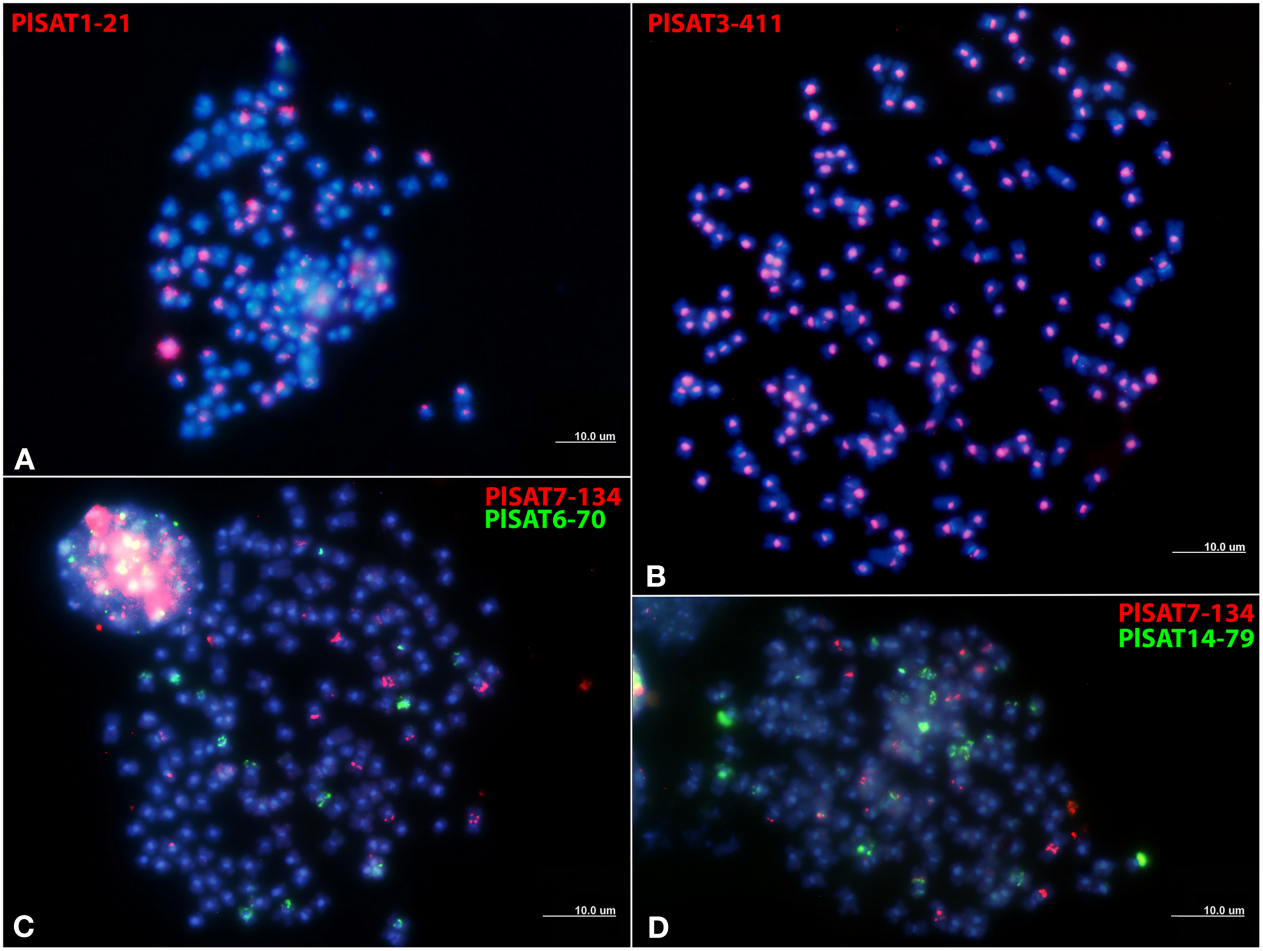
Distribution of satellite repeat families on metaphase chromosomes of *Pontastacus leptodactylus*. **(A)** PlSAT1-21 (in red), **(B)** PlSAT3-411 (in red), **(C)** PlSAT7-134 (in red) and PlSAT6-70 (in green), and **(D)** PlSAT7-134 (in red) and PlSAT6-70 (in green) as probes. Chromosomes are counterstained with DAPI. Scale bar = 10 μm.

Chromosome mapping of the PlSAT1-21, PlSAT3-411, PlSAT6-70, PlSAT7-134, and PlSAT14-79 satellites revealed distinct hybridization sites, with reproducible and unambiguous markings for all analyzed mitotic metaphases (Figure 4). PlSAT1-21 satellite family hybridized to the interstitial positions in the vicinity to the probable (peri)centromeric HSs on some chromosomes (Figure 4A). The PlSAT3-411 satellite hybridized in the (peri)centromeric regions, labeling all probable (peri)centromeric HSs on all *P. leptodactylus* chromosomes (Figure 4B). The PlSAT7-134 and PlSAT6-70 satellite families hybridized to the interstitial positions of some chromosomes (Figures 4C,D). The PlSAT14-79 satellite family co-localized with the AT-rich DAPI-positive probable (peri)centromeric heterochromatin on some chromosomes and is also located subterminally and intercalary on some chromosomes (Figure 4D). Besides, the PlSAT14-79 probe marked the whole shorter arm of one chromosome pair. The PlSAT6-70, PlSAT7-134, and PlSAT14-79 signals co-localized with some interstitial probable HSs.

### Similarity Between satDNA Families of *P. leptodactylus*

**Figure 5:**
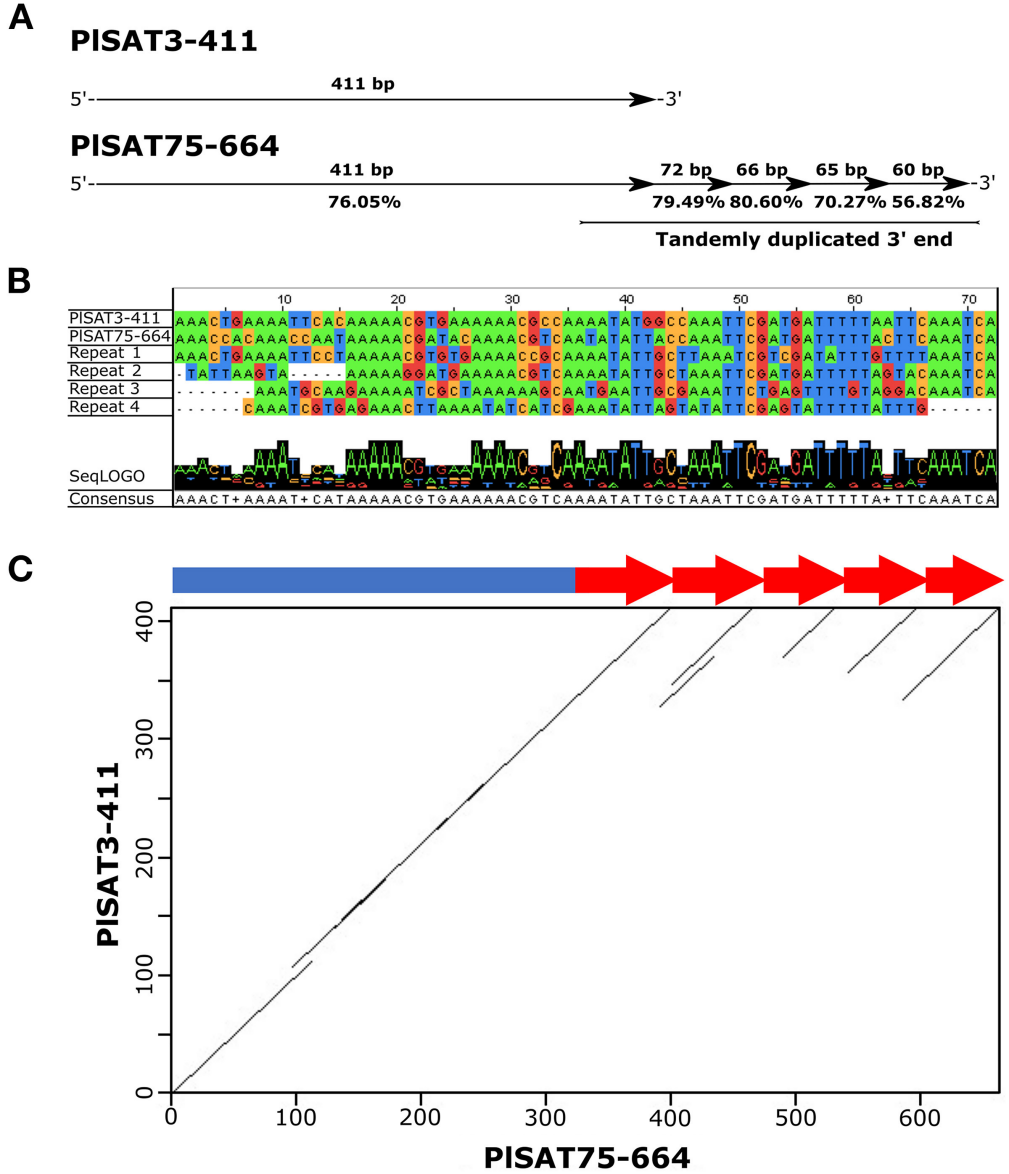
**(A)** Detailed analysis of satellite family PlSAT75-664 and its similarity with satellite family PlSAT3-411, **(B)** Alignment of PlSAT75-664 subrepeats visualized within Jalview (Waterhouse et al., 2009) and **(C)** Dot plot of the satellite family PlSAT75-664 obtained in the RepeatExplorer analysis of the *P. leptodactylus* genome, that shows similarity with the satellite family PlSAT3-411 satDNA of *P. leptodactylus*, revealing subrepeats with a periodicity of about 70 bp (arrows).

Some longer satDNA families showed similarity to other shorter families. Of 258 satellite repeats characterized in *P. leptodactylus*, 39 repeats showed similarities, forming 18 groups. Each group consisted of two or three satellite repeats. Similarity within each group ranged from 55 to 78%, average similarity is 63%. Only one satDNA family, PlSAT75-664, showed complex units including sub-repeats with high percentages of similarity to other shorter family, PlSAT3-411 (Figure 5). Detailed analysis showed that PlSAT75-664 unit includes the complete PlSAT3-411 unit and four direct sub-repeats, each ~70 bp long, each showing high similarity (79.9, 80.6, 70.21, and 56.82%) to 3′ end of the core of the PLSAT3-411 unit (Figure 5).

## Discussion

### Phylogenetic Placement of *P. leptodactylus* Individuals Used in This Study

Although*, Pontastacus leptodactylus* is naturally distributed across Europe, previous study by Njegovan et al. (2017) indicated that its presence in Lake Maksimir is the consequence of human mediated translocation. Phylogenetic reconstruction indicated that the individuals of *P. leptodactylus* belong to the Asian lineage *sensu* Maguire et al. (2014), specifically they are closely related to haplotypes originating from Armenia. Although, we observed two haplotypes within the Lake Maksimir population, they differed only in one base (site 378: Hap1-C, Hap2-A), collapsed to a single haplotype in the network reconstruction. This supports a theory by Njegovan et al. (2017) that the crayfish were introduced into lakes from the local market, supplied from the Armenian breeders. Further sampling and population studies, coupled with a multigene approach may help in resolving the taxonomic status of the three lineages within the *P. leptodactylus* species complex.

### *P. leptodactylus* Karyotype and Genomic Organization of 45S rDNA and Telomeric (TTAGG)*_*n*_* Repeats

In this study, FISH results showed one 45S rDNA locus and ten probable interstitial HSs in the studied *P. leptodactylus*, which is different from the previous work on *P. leptodactylus* that reported two 45S rDNA loci and six interstitial HSs (Mlinarec et al., 2011). The observed discrepancy suggests the presence of intraspecific variability within *P. leptodactylus*, and we could speculate that differences in rDNA loci number as well as in the number of interstitial HSs could possibly be lineage specific. In particular, samples analyzed in Mlinarec et al. (2011) belonged to European lineage *sensu* Maguire et al. (2014), while samples used in the present study belong to the Asian lineage *sensu* Maguire et al. (2014). Intraspecific variability has been reported in other groups of organisms such as two fish species from genus *Schistura* (Sember et al., 2015), as well as in plants *Phaseolus vulgaris* and *Tanacetum cinerariifolium* (Pedrosa-Harand et al., 2006; Mlinarec et al., 2019). Different mechanisms can lead to intrachromosomal variability such as unequal crossing-over, non-homologous recombination and movement mediated by transposons (Liu et al., 2003; Nguyen et al., 2010; Pereira et al., 2013; Vershinina et al., 2015; Mlinarec et al., 2019).

Large AT-rich probable HSs positioned in the (peri)centromeric position on all chromosomes and interstitially on some chromosomes suggest a high amount of repetitive DNA in the genome of *P. leptodactylus* (this study; Mlinarec et al., 2011). Large (peri)centromeric HSs have been found in different crustacean families such as Astacidae (Mlinarec et al., 2011, 2016), Nephropidae (Deiana et al., 1996; Coluccia et al., 2001; Salvadori et al., 2002), Scyllaridae (Deiana et al., 2007), Palinuridae (Coluccia et al., 1999, 2005; Cannas et al., 2004), Cambaridae (Salvadori et al., 2014), and Palaemonidae (González-Tizón et al., 2013; Torrecilla et al., 2017; Molina et al., 2020).

In this study it was observed that telomeres of *P. leptodactylus* consist of (TTAGG)_*n*_ pentameric repeats, same as in all decapod crustaceans studied until now and in most arthropods (Vítková et al., 2005; Salvadori et al., 2012, 2014). However, this study showed that a significant part of telomeric repeats is located interstitially in the chromosomes of *P. leptodactylus*. ITRs were also observed in other crustaceans such as *Jasus lalandii* and *Procambarus clarkii* (Salvadori et al., 2012, 2014). In *J. lalandii*, ITRs are associated with rDNA (Salvadori et al., 2012), while in *P. leptodactylus* and *P. clarkii* co-localization of ITRs with rDNA loci has not been observed (Salvadori et al., 2014). The occurrence of ITRs outside of the chromosomal termini is not fully understood. ITRs in (peri)centromeric regions could represent remnants of structural chromosome fusions (Ruiz-Herrera et al., 2008; Bolzán, 2012). This is unlikely in *P. leptodactylus* as there were no ITRs in (peri)centromeric positions. ITRs might have originated from the transposition of telomeric repeats by transposable elements or during repair of double stranded breaks (Aksenova and Mirkin, 2019) or might simply reflect the fact that telomeric sequences are present within repetitive DNA components like in some plants (Tek and Jiang, 2004; Mlinarec et al., 2009; Emadzade et al., 2014). The last case is unlikely as in *P. leptodactylus*, satellite repeats do not contain stretches of telomeric repeats.

### *Pontastacus leptodactylus* Repeatome

This work represents the most comprehensive characterization of the repetitive elements in any species belonging to the family Astacidae. In this study, we showed that *P. leptodactylus* harbors a large variety of repetitive elements, accounting for about 54.85% of its genome. As repeats may escape their detection by degradation, we consider this value as an underrepresentation. Degraded repeats arise from point mutations, indels and rearrangements, and they may be so substantial that they contribute repeats into tracks of unique or low-copy sequences. This is supported by recent studies on 101 species showing that in the large genomes, such as the genome of *P. leptodactylus*, the proportion of single and low-copy (up to 20 copies) sequences significantly increases with genome size, which is accompanied by a significant decrease in the genome proportion of medium-copy repeats (Novák et al., 2020a).

The analyses of draft genomes of *C. quadricarinatus* and *P. virginalis* showed that they have a significantly lower amount of repetitive DNA, 33.73 and 27.52%, respectively (Gutekunst et al., 2018; Tan et al., 2020), in comparison with *P. leptodactylus*. Furthermore, in *P. leptodactylus* satellite repeats and Ty3/*gypsy* elements are the most abundant, while in *C. quadricarinatus* and *P. virginalis*, LINE elements are the most abundant repetitive elements in the genome (Tan et al., 2020). However, comparison of the results of this study with those of Tan et al. (2020) should be taken with caution since different methods have been applied for repeat identification. Estimation of the repeat abundance form the *de novo* genome assemblies generated by short-read sequencing as in Tan et al. (2020), can lead to the underrepresentation of the highly repetitive elements. These elements are often clustered into a single contig or fragmented across multiple short contigs due to the inherited characteristics of the *de novo* genome assembly tools, therefore misrepresenting the abundance of the repetitive elements in the genome (Chu et al., 2016). The flow cytometry method estimated 1C = 18.7 Gbp size for the *P. leptodactylus* genome, providing the first report on genome size for any species within the family Astacidae. However, there is still a general lack of genome sizes for the infraorder Astacidea. As far as we are aware, genome size is available for several members of the familiy Cambaridae (5 species) and Parastacidae (1 species) ranging from 3.82 to 6.06 Gbp (Gregory, 2020; Tan et al., 2020). This makes *P. leptodactylus* (1C = 18.7 Gbp) species with the highest genome size of all known members of the infraorder Astacidea. In *P. leptodactylus*, genome expansion can be a result of the accumulation of short tandem repeats and retroelements as it is shown in this study that the genome of this species is rich in satellite DNA and retroelements. A large genome size as well as a highly repetitive genome explains difficulties generated during the genome assembly process, which limit the generation of available genomic resources from crustacean species (Tan et al., 2020; Van Quyen et al., 2020).

In *P. leptodactylus*, satellite repeats are the most abundant group of repetitive elements, accounting for 27.52% of its genome. Although, the knowledge about repetitive DNA composition in the genomes of decapod crustaceans is scarce, it is likely that a great expansion of satellites occurred in the genome of *P. leptodactylus*. The large amount of satellite repeats has been reported in other organisms such as insects *Drosophila virilis* and *Triatoma infestans* (Wei et al., 2014; Pita et al., 2018). In *D. virilis* nearly 50% of the genome is composed of satDNA, while in *T. infestans* satellite repeats make up 25-33% of the genome and are arranged into at least 42 satellite DNA families (Wei et al., 2014; Pita et al., 2018). Furthermore, we found great diversity of satDNA repeats in the genome of *P. leptodactylus* with a total of 258 satellite families which is by far the most satellite-rich species discovered to date. A large number of different satDNA elements is found in other organisms such as the fish *Megaleporinus macrocephalus* (Teleostei, Anostomidae) where 164 satellite repeats have been described (Utsunomia et al., 2019). Similar to *P. leptodactylus*, in *M. macrocephalus*, short satellites dominate in the genome. Among plants, *Luzula elegans* (Poaceae) has the highest number of satellites, 37, constituting 9.9% of the genome (Heckmann et al., 2013). The species *Vicia faba* (Fabaceae) is another example of the plant species with a high number of satellites, over 30, that together constitutes 935 Mbp (7%) of its genome (Ávila Robledillo et al., 2018). Large satDNA abundance and diversity is not a common characteristic for all animal and plant genomes, as there are, as far as we know, many more reports on the organisms poor in satellite DNA using similar approaches. In *Tanacetum cinerariifolium* (Asteraceae), only three among the 58,204 clusters obtained were classified as satellites, representing 1.04% of the genome (Mlinarec et al., 2019). Similarly, after the investigation of *Passiflora edulis* by RepeatExplorer, only two of the 233 repetitive elements were satellites, representing less than 0.1% of the genome (Pamponét et al., 2019).

It is tempting to speculate where the diversity of *P. leptodactylus* satellites originate from. Novel satellite DNA families may arise from the independent duplication of different genomic sequences, such as intergenic spacers, or even from those derived from other satellite DNAs (Garrido-Ramos, 2017). The satDNA sequences can interact with transposable elements to create new repetitive DNA (Pita et al., 2018). It is suggested that transposable elements provide the mechanism by which satDNA repeats could propagate in the genome through dispersed short repeat arrays (Macas et al., 2011; Bardella et al., 2014). The *P. leptodactylus* genome is rich in the LTR retrotransposons.

Minisatellites (monomer size 10–100 bp) were found to be surprisingly numerous in the *P. leptodactylus* genome, accounting for about 24.7% of the genome. High content of minisatellites in the *P. leptodactylus* genome might indicate a high level of DNA polymerase slippage as it is generally considered that short tandem repeats (<100 bp) expand through DNA polymerase slippage (Garrido-Ramos, 2017). The most abundant satDNA in the genome of *P. leptodactylus* is a minisatellite PlSAT1-21. Its short monomer size of 21 bp is unusual for a tandem repeat of high abundances, which generally consist of 160–180 or 320–360 bp monomers (Garrido-Ramos, 2017). This underpins that satellites with short monomer lengths can form very large arrays as observed here for PlSAT1-21. In the hermit crab *Pagurus pollicaris*, a minisatellite AGTGCAG(CTG)_n_ constitutes a large fraction of its genome (Chambers et al., 1978). An exceptional abundance of microsatellite and SSR sequences has also been found in the genome of freshwater prawns of the genus *Macrobrachium* (Palaemonidae) as well as in the penaeid shrimp *Litopenaeus vannamei* (Zhang et al., 2019; Molina et al., 2020), suggesting that short tandem repeats are a significant component of decapod crustaceans genomes.

In *P. leptodactylus*, 171 (66.27%) satellite DNA families showed A+T content higher than 50%, and could be classified as AT-rich (Figure 3). Furthermore, there is no correlation between A+T content and satellite length. The high A+T content could be a consequence of satDNA being subject to epigenetic modifications such as the methylation of cytosines, consequently deamination of 5-methylcytosines forming more AT base pairs in *P. leptodactylus* satDNAs. In the fish *Megaleporinus microcephalus* short (<100 bp) and long (>100 bp) satellites had a similar amount of A+T content (Utsunomia et al., 2019). In the fern *V. speciosa* satDNAs longer unit length showed a higher A+T content (Ruiz-Ruano et al., 2019). In *V. faba*, most of the satellite sequences had an elevated A+T content (65–80%) (Ávila Robledillo et al., 2018).

In *P. leptodactylus*, the satellites are abundant in the (peri)centromeric region, on both ends of the chromosomes and some of them are distributed on the interstitial regions of the chromosomes. This is in line with previous results which show that subtelomere and centromere regions contain large parts of satellite repeats (Melters et al., 2013; Garrido-Ramos, 2017). Conventional satellites (monomer size>100 bp) and minisatellites (monomer size 10–100 bp) are conventionally differentiated by their location (Garrido-Ramos, 2017). While classic satDNAs are usually located as long arrays at the heterochromatin segments, minisatellites are generally proper of euchromatic regions (Garrido-Ramos, 2017). In *P. leptodactylus*, the classic satellite family PlSAT3-411 constitutes (peri)centromeric HSs, while minisatellites PlSAT6-70 and PlSAT14-79 as a part of euchromatic regions are located along the chromosome arms.

### (Peri)centromeric Satellite Family PLSAT3- 411

Centromeres are often packaged into heterochromatin, containing large amounts of repetitive DNA (Wang et al., 2009; Mehta et al., 2010). Here we showed that the probable (peri)centromeric heterochromatic segments located on all *P. leptodactylus* chromosomes are formed by a specific highly amplified satellite family PlSAT3-411. The arrangement of the (peri)centromeric satDNA family PlSAT3-411 can be explained by the principle of equilocality, according to which, heterochromatin accumulates at equivalent positions in each chromosome within a genome (Garrido-Ramos, 2017). The most consistent form of equilocality relates to the heterochromatin in the vicinity of centromeres (John et al., 1985), which is true for PlSAT3-411 being present in the (peri)centromeric regions of all chromosomes. Following the survey of tandem satellite repeats in 282 species from various kingdoms (Melters et al., 2013), PlSAT3-411 is an ideal candidate for centromeric repeat sequences. It is one of the most abundant satellite repeats accounting for 1.29% of the genome and it is A+T-rich. It has been found that centromeric satDNAs are generally A+T rich (Garrido-Ramos, 2015; Yuan et al., 2018). Most animal species investigated so far have a single or only a few centromeric satellites with monomers hundreds of nucleotides long that are shared by all chromosomes, an observation that is explained by their coevolution with kinetochore proteins (Garrido-Ramos, 2015). The (peri)centromeric satellite family PlSAT3-411 is common in that respect. (Peri)centromere composition of *P. leptodactylus* calls for the investigation of additional species from different genera to get a more representative insight into the evolution of the (peri)centromeric satellite family. The frequent accumulation of satDNA in centromeric regions is explained by its role in centromere functions, such as kinetochore assembly and chromosome segregation during mitosis or meiosis, or even some epigenetic regulations, or simply by passive accumulation due to the absence of recombination-based elimination mechanisms (McFarlane and Humphrey, 2010; Plohl et al., 2014; Catania et al., 2015). To fully confirm that PlSAT3-411 is a true centromeric satellite family, underlying the functional kinetochore CENH3-ChIP followed by sequencing is needed.

### Similarity Between satDNA Families

Most of the satDNA families described in this study did not show any conserved features or sequence similarities between each other suggesting their independent origin. Only 39 of the 258 satDNA repeats described in *P. leptodactylus*, showed similarities, however, their similarity is not high, ranging from 55 to 78%, average similarity is 63%. Two satellites, PlSAT3-411 and PlSAT75-664, were among the most interesting. The longer unit PlSAT75-664 is organized into HOR (higher order repeat) structures that consist of PlSAT3-411 basic monomer and four times directly repeated ~70 bp long sequence that shows high similarity to PlSAT3-411 (Figure 5). The similarity between PlSAT3-411and PlSAT75-664 indicates the existence of a satDNA superfamily (SF), derived from a common ancestor satDNA. In the most parsimonious scenario, HOR structure might have formed after a ~70 bp fragment was four times amplified within the satDNA, resulting in a new repeat unit of 664. It is known that the simultaneous amplification and homogenization of two or more adjacent monomers leads to the formation of HORs (Garrido-Ramos, 2017). Furthermore, it is generally considered that shorter repeats originate by replication slippage, while longer units originate by unequal crossing over (Garrido-Ramos, 2017). Therefore in *P. leptodactylus*, replication slippage might be the mechanism for the origin of the four times tandemly repeated ~70 bp subunits within PlSAT75-664. The combination of short repeat units into longer units constituting HORs is a common trend in satDNA evolution (Plohl et al., 2008; Garrido-Ramos, 2017). Regular HORs, usually dimeric, have been found in several species of beetles (Palomeque and Lorite, 2008; Vlahović et al., 2017). Complex HORs, shaped from interspersed and/or inversely oriented monomers and frequently with extraneous sequence elements, have been found in non-human mammals, such as mouse, pig, bovid, horse, dog, elephant, insect, and fish (Palomeque and Lorite, 2008; Vlahović et al., 2017; Utsunomia et al., 2019).

The present study is the first one focusing on the repeatome of *P. leptodactylus* and enables a new perspective into the evolution of this complex species. *P. leptodactylus* repeatome serves as an important and valuable resource to support ongoing comparative genomic, cytogenomic, fundamental, and applied biology studies. To gain a more comprehensive understanding of chromosome evolution and genomic compositions of freshwater crustaceans, chromosome and genome resources are much needed for more species across taxonomic groups.

## Data Availability Statement

Publicly available datasets were analyzed in this study. This data can be found here: https://www.ebi.ac.uk/ena/browser/view/SRR7698976, SRR7698976; https://www.ncbi.nlm.nih.gov/nuccore/KX279350.1, KX279350.

## Author Contributions

The study was conceived by IM, VB, JM, LLB, and LB, while LLB, LB, and JM designed the experimental part of the study. Field work was carried out by LLB, LA, LB, and LL, while lab work (DNA isolation, cytogenetic experiments) by LLB, LA, LB, LL, and IM. CG and AH conducted flow cytometry analysis. LLB carried out the bioinformatic analyses and JM designed the primers. JM, LLB, LB, IM, VB, FG, and CA discussed and interpreted the results. JM wrote the paper with the help of LLB and LB. The initial version of the manuscript was drafted by IM, VB, LLB, and LB. All authors read, edited, enhanced original version of the manuscript, and approved its final version.

## Conflict of Interest

The authors declare that the research was conducted in the absence of any commercial or financial relationships that could be construed as a potential conflict of interest.

## Supplementary Material

Supplementary Table 1 *COI* gene sequences from *P. leptodactylus* used to construct a phylogenetic tree.

Supplementary Table 2 *Pontastacus leptodactylus* satellitome. Repeat unit lengths, G+C content (%), abundances (%), and consensus sequence.

Supplementary Table 3 Morphometric measurements of *P. leptodacylus* chromosomes (*n* = *x* = 90).

Supplementary Figure 1 Consensus sequences of PlSAT1-21, PlSAT3-411, PlSAT6-70, PlSAT7-134, and PlSAT14-79 repeats in fasta format. Primer sequences for amplification of tandem repeat specific probes are underlined and bolded.

Supplementary Figure 2 Median-joining network of the *COI* barcode region haplotypes. Haplotype from the lake Maksimir is marked with an arrow. Haplotype size reflects relative frequency. Each branch represents one mutational step, unless otherwise noted (numbers in red above branches). Black circles represent missing intermediate haplotypes. Different colored circles denote the share of distinct haplotypes within countries (legend is shown in the upper-left corner).

Supplementary Figure 3 An idiogram of *P. leptodactylus* chromosomes with marked localization of 45S rDNA and DAPI positive AT-rich heterochromatin bands This idiogram was generated based on the FISH information from Figures 1A,B.

Supplementary Figure 4 The graph layout corresponding to read clusters of **(A)** PlSAT1-21, **(B)** PlSAT3-411, **(C)** PlSAT6-70, **(D)** PlSAT7-134, and **(E)** PlSAT14-79. The percentage indicates the genome proportion of each cluster.

Supplementary Figure 5 Sequence logos showing the level of sequence divergence.

Supplementary Figure 6 Dot plot analysis of **(A)** PlSAT1-21, **(B)** PlSAT3-411, **(C)** PlSAT6-70, **(D)** PlSAT7-134, and **(E)** PlSAT14-79 obtained in TAREAN analysis of *P. leptodactylus*.

## Abbreviations

FISH: Fluorescence *in situ* hybridization
HOR: higher order structures
HSs: heterochromatin segments
ITRs: interstitial telomeric repeats
LTRs: long terminal repeats
rDNA: ribosomal DNA
SFs: superfamilies
SSRs: simple sequence repeats
TEs: transposable elements.
