# Supplementary material for: The Pontastacus leptodactylus (Astacidae) Repeatome Provides Insight Into Genome Evolution and Reveals Remarkable Diversity of Satellite DNA
Source: Front Genet. 2021 Jan 21;11:611745. doi: 10.3389/fgene.2020.611745 (PMC7859515; doi:10.3389/fgene.2020.611745)
Supplement: Supplementary Figure 1 — Consensus sequences of PlSAT1-21, PlSAT3-411, PlSAT6-70, PlSAT7-134, and PlSAT14-79 repeats in fasta format. Primer sequences for amplification of tandem repeat specific probes are underlined and bolded. [file Image_1.pdf]

PlSAT1-21-dimer
>**AGTTTCAATCGTCCCTGCTG**AA**GTTTCAATCGTCCCTGCTGA**

PlSAT3- 411-monomer
>CGTTTATACAATATCTCACTTAGTTTTT**GAACGAAATGCAAATGGTTGAT**CAAAA
ATTATGTATTTACGCTAAATCTACAATATAATATGATTTTGAACCATATTTATCGTAT
ATTTAATGAATTAATAACATTAAACTGAAAATTCACAAAAACGTGAAAAAACGCCA
AAATATGGCCAAATTCGATGATTTTTAATTCAAATCACAAAATGAGATTTCGGCTTA
TTTATAGCATTAAAATACATATGAAGATTTAGTTTCCAATTCAAATGATCAAATTCA
ATGTGTTTTCATAAGATTTAACCATAATAT**TGTCTATTTTCCGTATATTGTAATGA**T
AATACAAGTACGCGTAAAACAGCTCCAAACCGGCCTGAAACGGCGAAATATGCAAT
AAAAACATGCTTTTT

PlSAT6- 70-monomer
>**ACTTACCTTGCAGGCACATATA**GGTGAGAACAACTACATATA**GACATGTTTTAC
ATTAGACTTGTGA**GAA

PlSAT7- 134-monomer
>AATTTTTTTTTTAAAACGTTACCCAAATCCTC**GTCTACTTTCGTTACGGA**TAAAGA
TTATTCAAATATTTTTCTAGAGTAATATT**GGCAAGCCCAATTGGGTCTGA**TTGCAT
ACCAAAAAAGTTGAAATATATT

PlSAT14- 79-monomer
>**CCTTAATAACCTTCCATAGGTTG**ATAGGCGTTAAGCCCAACACCAAACTTGCTGG
TT**GGTCAGTAAGCTATTGTGTGT**G
