# Supplementary figures and images for: The Pontastacus leptodactylus (Astacidae) Repeatome Provides Insight Into Genome Evolution and Reveals Remarkable Diversity of Satellite DNA
Source: Front Genet. 2021 Jan 21;11:611745. doi: 10.3389/fgene.2020.611745 (PMC7859515; doi:10.3389/fgene.2020.611745)

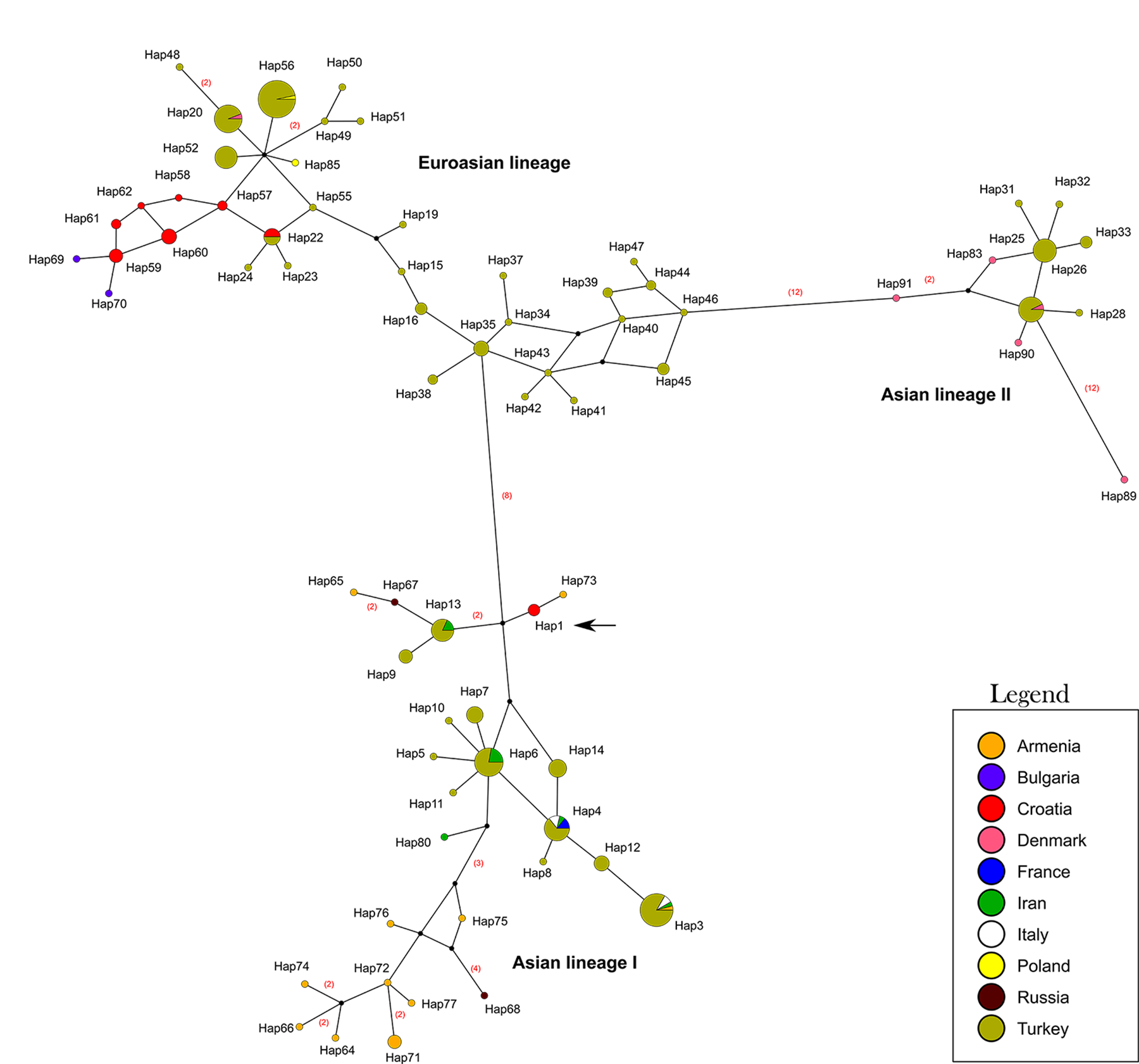

Supplement: Supplementary Figure 2 — Median-joining network of the COI barcode region haplotypes. Haplotype from the lake Maksimir is marked with an arrow. Haplotype size reflects relative frequency. Each branch represents one mutational step, unless otherwise noted (numbers in red above branches). Black circles represent missing intermediate haplotypes. Different colored circles denote the share of distinct haplotypes within countries (legend is shown in the upper-left corner). [file Image_2.tif]

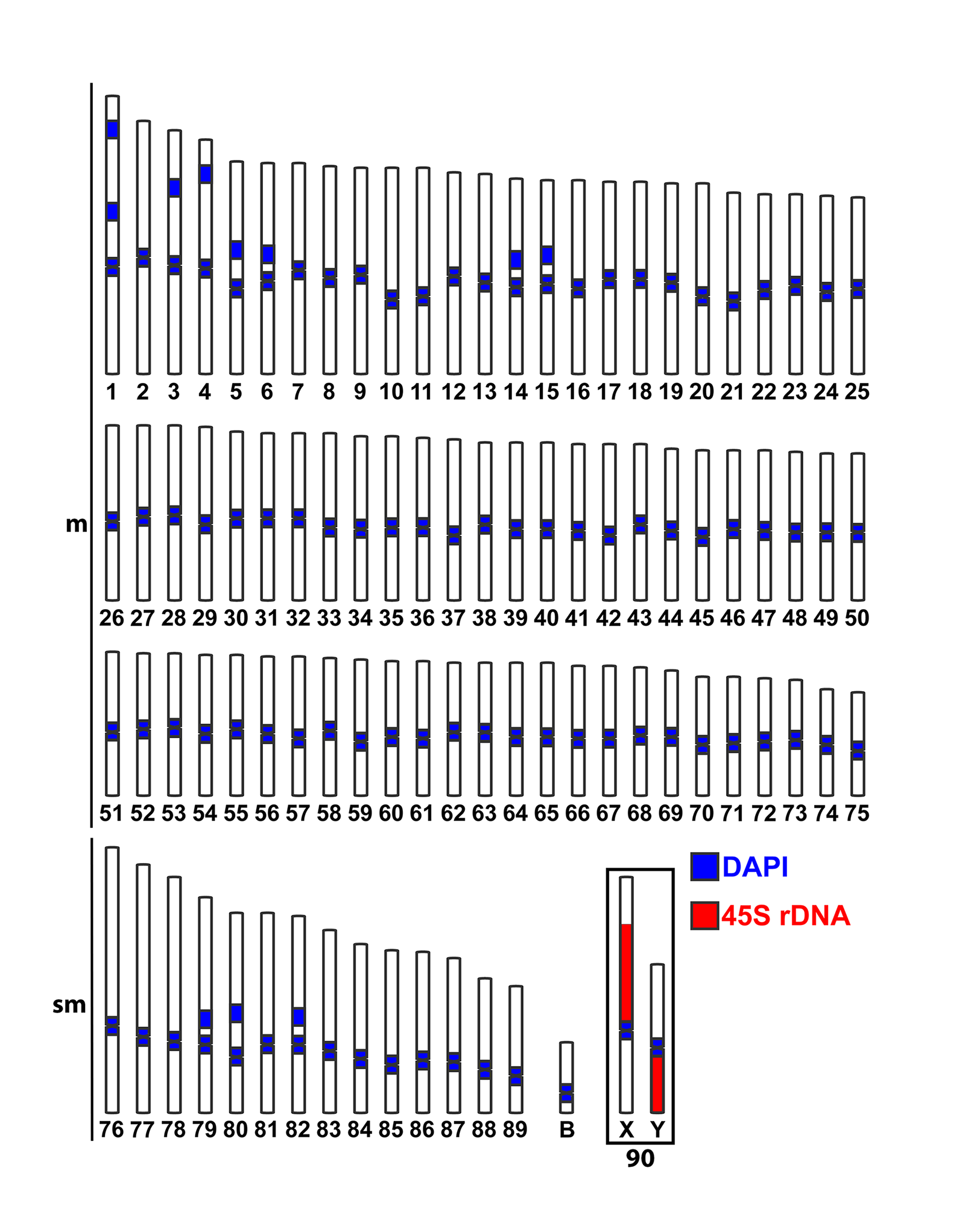

Supplement: Supplementary Figure 3 — An idiogram of P. leptodactylus chromosomes with marked localization of 45S rDNA and DAPI positive AT-rich heterochromatin bands This idiogram was generated based on the FISH information from Figures 1A,B. [file Image_3.tif]

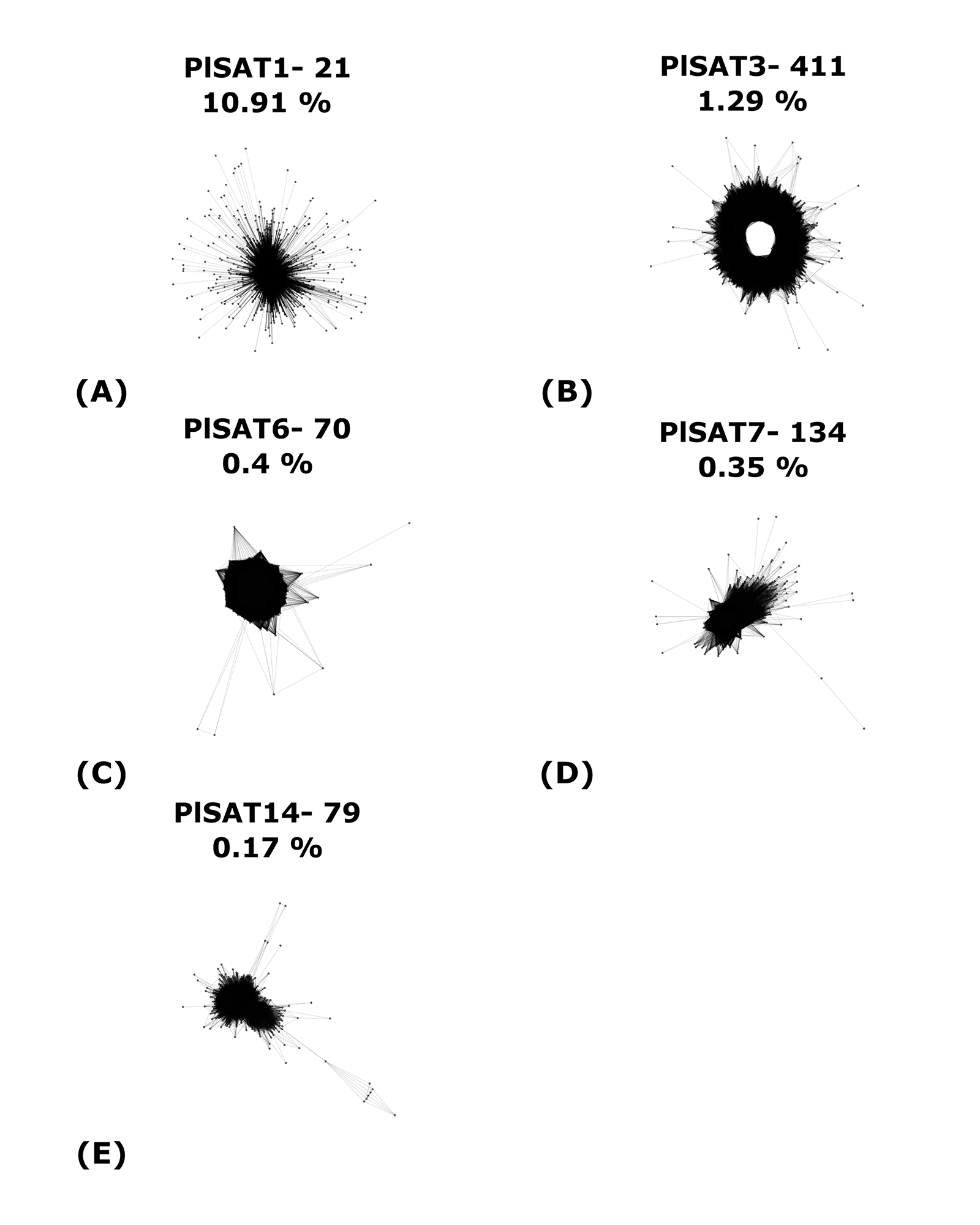

Supplement: Supplementary Figure 4 — The graph layout corresponding to read clusters of (A) PlSAT1-21, (B) PlSAT3-411, (C) PlSAT6-70, (D) PlSAT7-134, and (E) PlSAT14-79. The percentage indicates the genome proportion of each cluster. [file Image_4.tif]

PISAT1-21

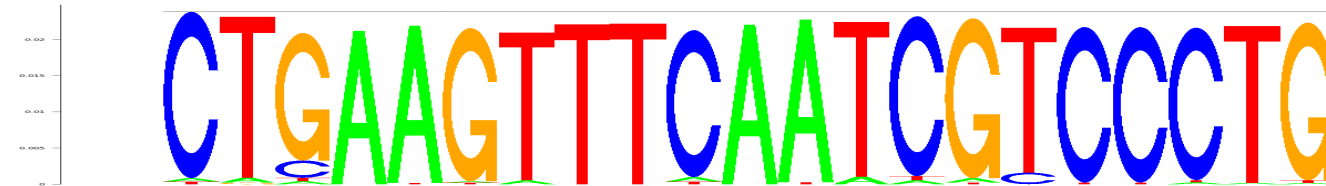

PISAT3-411

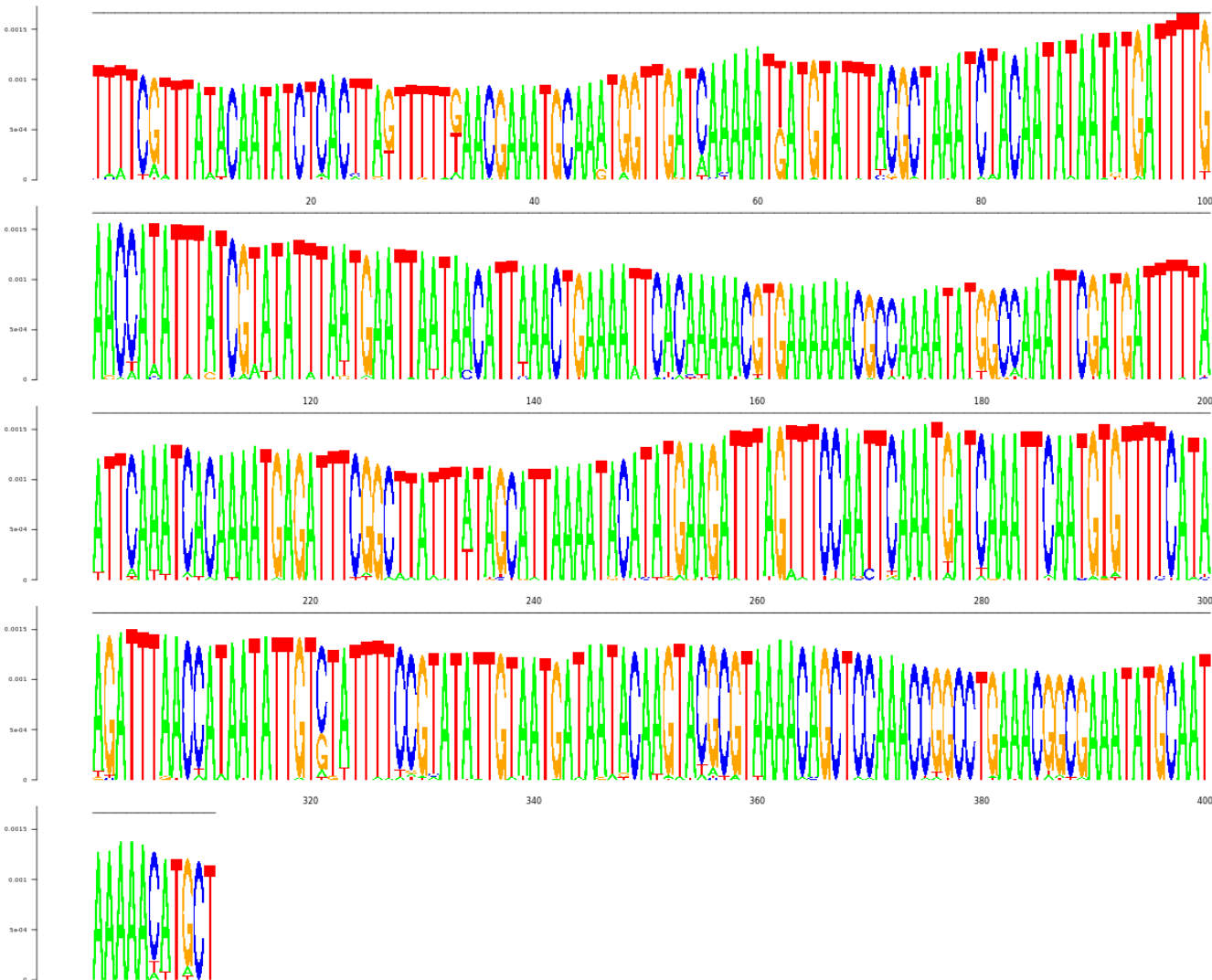

PISAT6-70

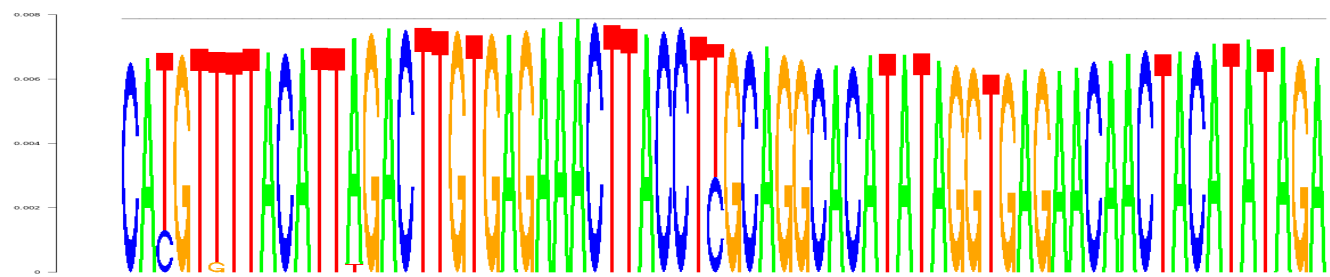

PISAT7-134

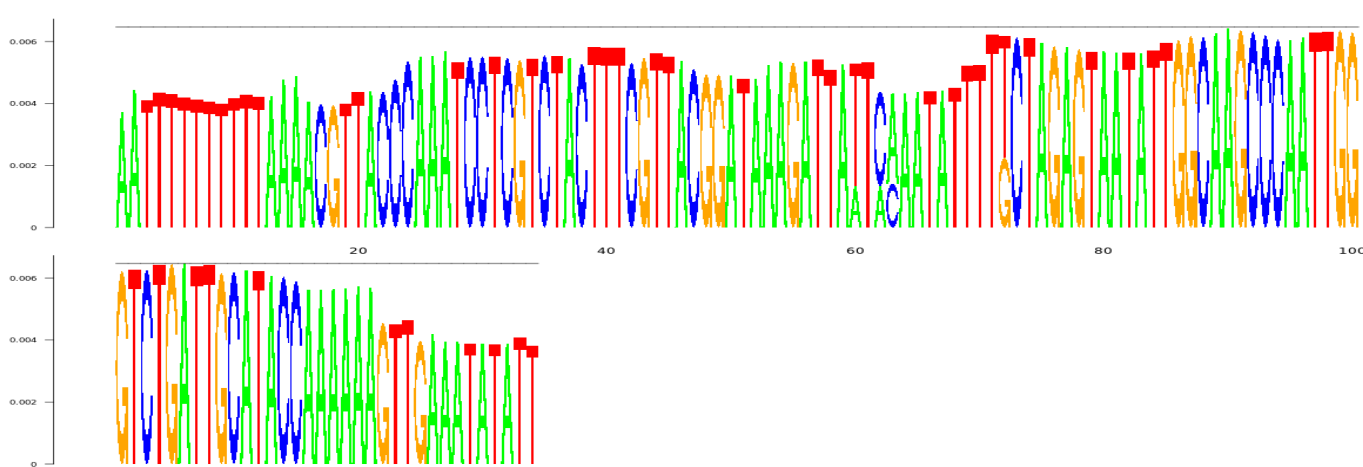

PISAT14-79

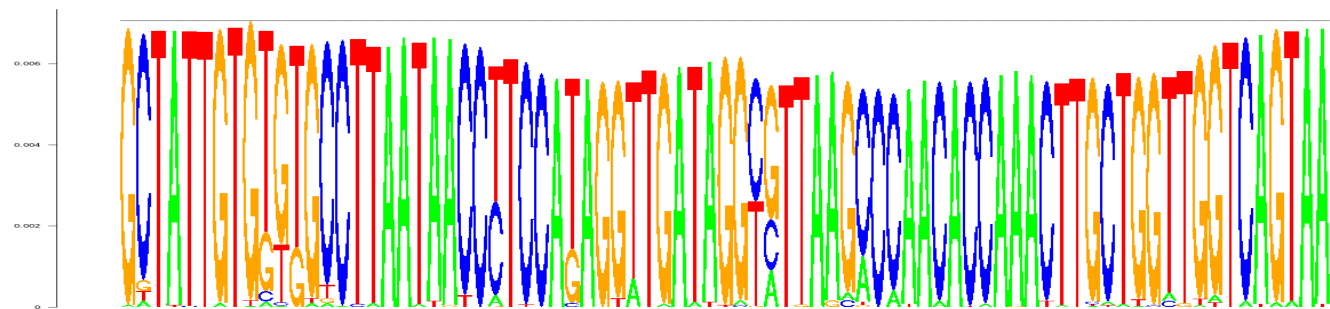

Supplement: Supplementary Figure 5 — Sequence logos showing the level of sequence divergence. [file Image_5.pdf]

**PISAT1-21**

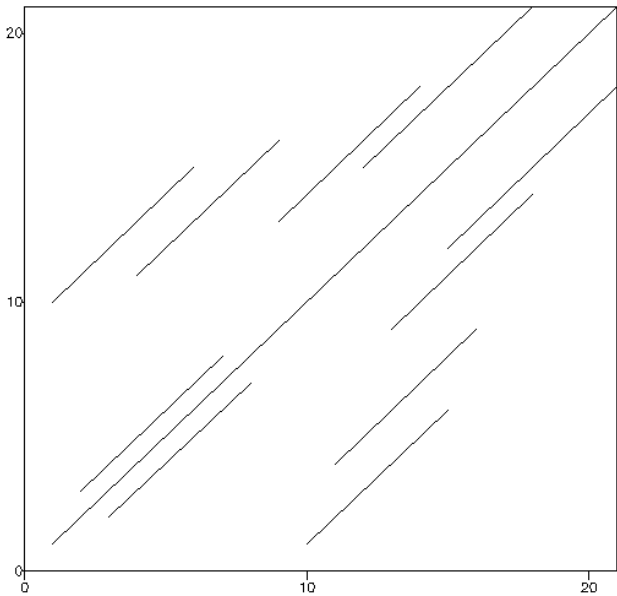

**(A)**

**PISAT3-411**

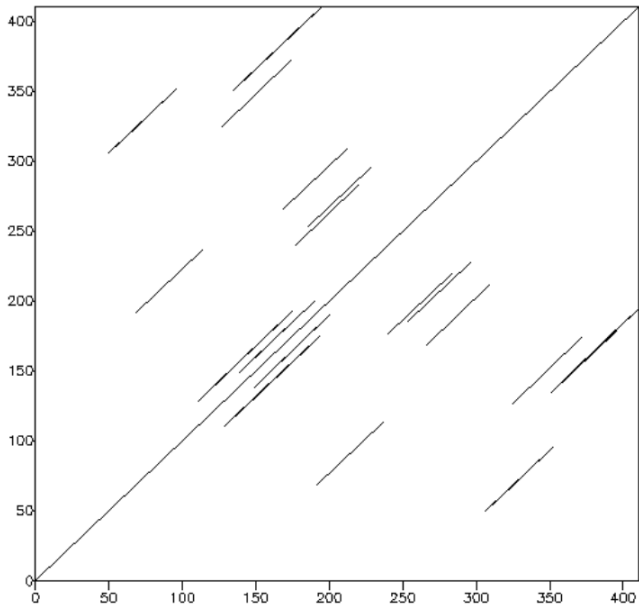

**(B)**

**PISAT6-70**

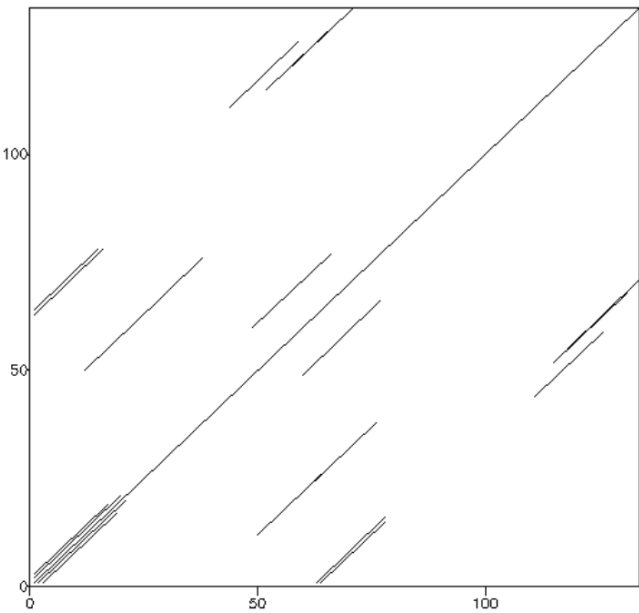

**(C)**

**PISAT7-134**

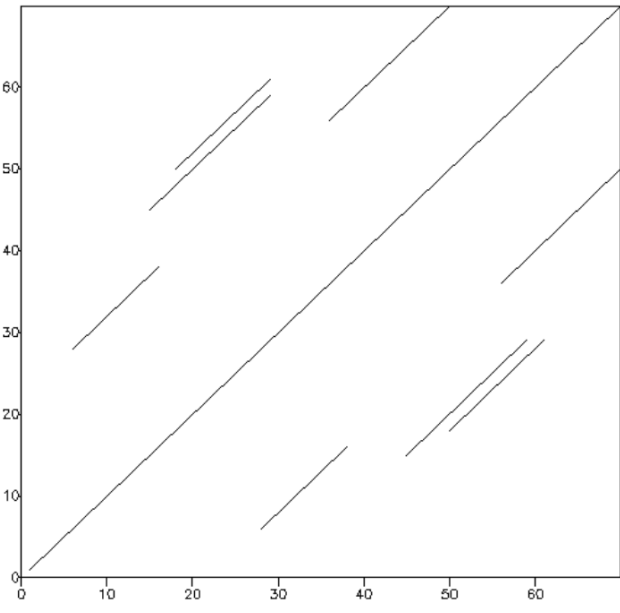

**(D)**

PISAT14-79

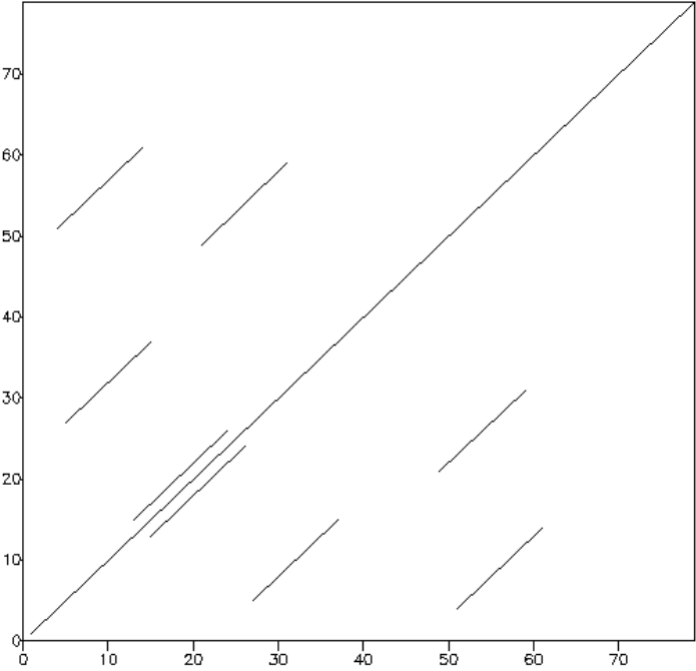

(E)

Supplement: Supplementary Figure 6 — Dot plot analysis of (A) PlSAT1-21, (B) PlSAT3-411, (C) PlSAT6-70, (D) PlSAT7-134, and (E) PlSAT14-79 obtained in TAREAN analysis of P. leptodactylus. [file Image_6.pdf]
